# Praseodymium effect on growth and antioxidant systems in *Lemna minor*

**DOI:** 10.7717/peerj.21790

**Published:** 2026-11-09

**Authors:** Rolandas Karitonas, Brigita Gylytė, Vira Khoma, Jūratė Vaičiūnienė, Oksana Stoliar, Levonas Manusadžianas

**Affiliations:** 1 State Scientific Research Institute Nature Research Centre, Vilnius, Lithuania; 2 Ternopil Volodymyr Hnatiuk National Pedagogical University, Ternopil, Ukraine; 3 Institute of Chemistry, Centre for Physical Sciences and Technology, Vilnius, Lithuania

**Keywords:** Praseodymium, *Lemna minor*, Bioaccumulation, Oxidative stress, Antioxidants, Biotransformation enzyme activities

## Abstract

The increasing use of rare earth elements (REEs) elicits concerns about their release into freshwater environments and potential effects on primary producers. This study investigated the toxicity of praseodymium (Pr) to the aquatic macrophyte *Lemna minor* during a 7-day exposure, centring on growth inhibition, Pr accumulation, oxidative stress and responses of related enzymes. A tenfold increase in Pr concentration in the medium from 0.01 to 0.1 mM resulted in a 56-fold increase in Pr content in plant tissue, with a bioconcentration factor of 608 L/kg. Further increases (0.2–0.4 mM) worsened the plant’s physiological state, yielding less than a 50% additional increase in Pr content. No significant effects were observed at 0.01 mM Pr, whereas inhibition of root growth occurred at 0.1 mM and frond growth at 0.2 mM. At the highest tested concentration (0.4 mM), frond and root growth rates were reduced by 37% and 94%, respectively, and visible chlorosis developed from 0.1 mM onward. Pr induced oxidative stress in *L. minor*, as evidenced by significantly elevated hydrogen peroxide and malondialdehyde levels at 0.3–0.4 mM. These changes were accompanied by marked alterations in antioxidant and detoxification-related enzyme activities, including a 76% increase in glutathione S-transferase activity at 0.4 mM Pr. Overall, the combined physiological and biochemical data demonstrate that *L. minor* readily accumulates praseodymium, which induces pronounced growth inhibition and oxidative stress at elevated concentrations. These findings provide element-specific quantitative evidence of Pr toxicity in *L. minor*. The strong accumulation capacity of this duckweed indicates potential relevance for Pr removal from contaminated waters, while also showing a possible route for Pr, and presumably other REEs, to enter aquatic food webs.

## Introduction

Praseodymium (Pr), a rare earth element (REE), is increasingly utilised in permanent magnets (King & Eggert, 2022), advanced alloys for aircraft engines and turbines (Wang et al., 2016), optics (Abouhaswa, Rabiea & Abomostafa, 2023), catalysts, such as those for diesel soot oxidation (Li et al., 2023), and clean energy conversion (Khan et al., 2025). The expanding technological applications of Pr and other REEs raise concerns regarding their potential release into the environment and subsequent bioavailability to aquatic organisms.

Praseodymium has been detected in wastewater treatment plant (WWTP) discharges and associated rivers in the USA (Lee et al., 2011) and Canada (Turcotte et al., 2022), industrial effluents (Kulaksiz & Bau, 2011), the municipal sewage sludge (Kostrz-Sikora, 2024), and water bodies affected by acid mine drainage (Gomes et al., 2022; Viana et al., 2024). Reported concentrations of dissolved Pr are typically low, ranging from sub-µg/L levels in rivers and WWTP effluents to tens of µg/L in mine-impacted waters. Elevated concentrations are thus primarily associated with specific anthropogenic sources.

Existing toxicity data for primary producers are unevenly distributed across taxonomic groups. In algae, Tai et al. (2010) demonstrated that 13 lanthanides induced growth inhibition at nearly identical 96-h EC_50_ values of ∼30 µM in the diatom *Skeletonema costatum*, indicating a uniform response to the lanthanide series rather than element-specific sensitivity. Joonas et al. (2017) reported similar 72-h IC_50_ values, ranging from 9 to 10 µM (1.2–1.4 mg/L), for *Raphidocelis subcapitata* exposed to four lanthanides, including Pr. The 24-d LC_50_ for the macrophytic algae *Nitellopsis obtusa* exposed to PrCl_2_ was approximately 3.2 mg/L (∼22 µM) (Manusadžianas et al., 2020). In aquatic plants, Xu et al. (2016) found that 20–60 µM PrCl_3_ induced oxidative stress and photosynthetic impairment in *Spirodela polyrrhiza*. Recent studies have examined the effects of REEs in *Lemna minor*, exploring various toxicological aspects (Weltje et al., 2002; Zicari et al., 2018; Alp et al., 2023; Gjata et al., 2025); however, data on Pr toxicity in this duckweed species remain unavailable.

Given that environmentally measured dissolved Pr concentrations are generally much lower than levels expected to cause acute toxicity, this study employed a concentration range of 0.01–0.4 mM Pr to identify toxicity thresholds and characterise mechanistic effects based on physiological and biochemical responses under elevated exposure conditions. These concentrations represent high-exposure or worst-case scenarios, relevant to localised contamination events, rather than typical background freshwater exposure. Within this framework, the effects of Pr on the floating aquatic plant *Lemna minor* were assessed after 7 days of exposure by analysing growth, accumulation, oxidative stress markers, and alterations in antioxidant enzyme activities (catalase (CAT), guaiacol (GPOD) and pyrogallol (PPOD) peroxidases, ascorbate peroxidase (APX), dehydroascorbate reductase (DHAR), glutathione-S-transferase (GST)) as well as the ascorbate (AsA)/dehydroascorbate (DAsA) pool.

## Materials and Methods

### Plant material

We have been collecting duckweed (*Lemna minor* L.) plants from a local freshwater pond (54°75′N, 25°29′E; Verkiai Regional Park, Vilnius) and have been culturing fronds under controlled conditions for several years. The local freshwater pond is located in freely accessible publicly owned land, and no permission was needed to access it. The species specificity of the experimental clone, which was morphologically similar to *L. minor,* was previously confirmed by chloroplast DNA sequencing (Karitonas et al., 2020). A stock culture was maintained in 500-mL plastic containers (60 × 85 × 100 mm, WxLxH) in a modified Schenk and Hildebrandt growth medium (Schenk & Hildebrandt, 1972) without vitamins, myo-inositol and sucrose. The pH was adjusted to 6.8 with 1 M NaOH. Stock cultures and treated plants were maintained in growth chambers at 24 ± 2 °C under constant cool-white, fluorescent illumination at a photosynthetic photon flux density of 160 µmol m^−2^ s^−1^.

### Chemicals

Praseodymium chloride salt (PrCl_3_⋅H_2_O) was purchased from Sigma-Aldrich (PCode 1003669377; Burlington, MA, USA). The growth medium chemicals were from Merck (Darmstadt, Germany).

### Experimental design

The experimental scheme comprised control (growth medium) and five treatments (growth medium supplemented with 0.01, 0.1, 0.2, 0.3 and 0.4 mM Pr) for the growth experiments, and four treatments (0.1, 0.2, 0.3 and 0.4 mM Pr) for the H_2_O_2_, lipid peroxidation, antioxidant enzyme activities and ascorbate determination. Plants were incubated in 500-mL plastic containers (60 × 85 × 100) containing 200 mL of the corresponding medium. To avoid precipitation of Pr, ammonium phosphate in deionised water was added directly to the fronds. A similar procedure was applied to the controls to ensure that it did not affect duckweed development. The media were adjusted to pH 6.8. Solutions were not renewed during the 7-day exposure period.

For growth experiments, healthy colonies with 2–3 fronds from stock cultures were transferred into containers with the corresponding exposure medium. The number of fronds and root length were measured at 0- and 7-day time points using an image analysis system (MOTIC 2.0 software). The growth rate per day was calculated with the following equation: r = (lnx_t2_-lnx_t1_)/t_2_-t_1_, where x_t1_ and x_t2_are the values of the observation parameter at t_1_ and t_2_ days, respectively. The experiments were conducted in triplicate.

To explore H_2_O_2_, lipid peroxidation, ascorbate content, and antioxidant enzyme activities, the cultures were initiated by transferring healthy colonies with 3–4 fronds from stock cultures into four containers (0.8–0.9 g each) for each exposure medium. Plant samples were collected after 7 days of exposure. Four replicates were conducted for each parameter at each treatment concentration. Then, samples of *L. minor* (0.2 g each) were processed as described by Hildebrand et al. (1986) to determine CAT, GPOD and PPOD enzyme activities. Cold potassium phosphate buffer (0.1 M, pH 7.0) containing 1% (w/v) polyvinylpyrrolidone and 1% (v/v) Triton X-100 was added to a chilled (4 °C) mortar and pestle containing the sample.

For determination of APX, DHAR and GST activities, enzymes were extracted in two mL of 50 mM Tris–HCl, pH 7.0, containing 20%(v/v) glycerol, one mM AsA, one mM dithiothreitol (DTT), one mM ethylenediaminetetraacetic acid (EDTA), one mM glutathione (GSH) and five mM MgCl_2_ (Knörzer, Burner & Boger, 1996).

Each sample was macerated with one mL of the extraction buffer and was further ground with another one mL of the buffer. A two mL aliquot of homogenate was centrifuged at 13,000 rpm for 15 min at 4 °C (Hou et al., 2007). The supernatant was immediately frozen for subsequent total protein content and enzyme assays. Total protein content was determined using bovine serum albumin as a standard (Bradford, 1976). Spectrophotometric measurements were performed on a Libra S32 PC UV-Vis spectrophotometer (Biochrom, Cambridge, UK).

### Chlorophyll and carotenoid contents

Plant samples (0.010 g) were homogenised at 4 °C with 80% acetone 1:15 w/v, and homogenates were centrifuged at 10,000 g for 10 min. The absorbance at 663 nm for chlorophyll a, 646 nm for chlorophyll b and 470 nm for carotenoids was measured in the supernatant using a Biochrom Libra S32 PC spectrophotometer and the pigment contents were determined as reported by Lichtenthaler & Wellburn (1985).

### Determination of Pr

Before determining the metal content in the media or duckweed at the end of the 7-day exposure, the plants from the control and treatment groups (four replicates) were washed three times with deionised water. All liquids on the surface of plant materials were blotted with paper towels. 0.2 g of fresh plant material was transferred to a ceramic crucible (Haldenwanger, Waldkraiburg, Germany) in a muffle furnace (SNOL-1.6.2.5, Borispol, Kyiv, Ukraine) to thermally decompose the combustible (organic) portion of the sample at 450–550 °C for 2–3 h. After the sample was digested in 0.5 mL pure HNO_3_ (Roth, Karlsruhe, Germany) and heated until the acid evaporated to half its original volume, it was diluted to a final volume of five mL with dH_2_O.

The Pr content was determined using a PerkinElmer Optima 7000 Dual View ICP Optical Emission Spectrometer (Waltham, MA, USA) with the standard method and was calculated from the standard curve. Pr concentration was expressed as mg/L or µg/g fresh weight (FW).

### Determination of hydrogen peroxide

H_2_O_2_ levels were determined according to Alexieva et al. (2001) by measuring spectro-photometrically after reaction with potassium iodide (KI). Fresh plant material (0.2 g) was homogenised in an ice bath with two mL 0.1% (w/v) trichloroacetic acid (TCA). The homogenate was centrifuged at 13,000 rpm for 15 min, and 0.5 mL of supernatant was added to 0.5 mL of 10 mM potassium phosphate buffer (pH 7.0) and one mL of 1 M KI. The absorbance of the supernatant was read at 390 nm. The H_2_O_2_ content was measured at 390 nm and then calculated from a standard curve.

### Determination of lipid peroxidation

The level of lipid peroxidation in the plant was assessed by thiobarbituric acid (TBA) reactive metabolites, chiefly malondialdehyde (MDA), as described by Heath & Packer (1968). Plant tissues (0.2 g) were extracted in two mL of 0.25% TBA made in 10% TCA. The extract was heated to 95 °C for 30 min and then quickly cooled on ice. After centrifugation at 13,000 rpm for 10 min, the absorbance of the supernatant was measured at 532 nm. Non-specific turbidity was corrected by subtracting the absorbance at 600 nm. The level of lipid peroxidation was expressed as MDA concentration formed using *ɛ* = 155 mM^−1^ cm^−1^.

### Enzyme assays

CAT (EC 1.11.1.6) activity was determined by measuring the decrease in H_2_O_2_ in the reaction mixture at 410 nm using a spectrophotometer, as described in Koroliuk et al. (1988). One unit of CAT activity was defined as the amount of enzyme required to decompose one µmol H_2_O_2_ per min (μkat) per g of protein.

Peroxidase (POD, EC 1.11.1.7) activity was assayed by the method of Chance & Maehly (1954), using either guaiacol or pyrogallol as the reductant. GPOD activity was measured by following the increase in absorbance at 470 nm, due to the formation of tetraguaiacol (*ɛ* = 26.6 mM^−1^ cm^−1^). The reaction mixture contained 50 mM potassium phosphate (pH 7.0), 0.25% (18 mM) guaiacol, 5 mM H_2_O_2_, and extract, for a final volume of one mL, at 25 °C. The PPOD assay mixture contained 20 mM pyrogallol, one mM H_2_O_2_ and enzyme in the same buffer. Purpurogallin formation was measured at 430 nm (*ɛ* = 2.47 mM^−1^ cm^−1^).

APX (EC 1.11.1.11) was assayed as the decrease in absorbance at 290 nm resulting from AsA oxidation, by the method of Nakano & Asada (1981). The reaction mixture contained 50 mM potassium phosphate, pH 7.0, one mM Na ascorbate, 2.5 mM H_2_O_2_ and enzyme in a final volume of one mL at 25 °C.

DHAR (EC 1.8.5.1) was assayed directly by following the formation of AsA at 265 nm, according to Hossain & Asada (1984). The reaction mixture contained 50 mM potassium phosphate, pH 7.0, 0.5 mM DAsA, 2.5 mM GSH and enzyme in a final volume of one mL at 25 °C

GST (EC 2.5.1.18) was assayed as the increase of absorbance at 340 nm due to the conjugation of GSH to 1-chloro-2,4-dinitrobenzene (CDNB), as described by Drotar, Phelps & Fall (1985), in 100 mM potassium phosphate, pH 7.0, containing two mM CDNB, two mM GSH and enzyme.

### Extraction of ascorbate

Plant tissues (0.5 g) were homogenised with a mortar and pestle in ice. Subsequently, ascorbate was extracted by grinding the powder with two mL of 5% (w/v) metaphosphoric acid. Centrifugation was carried out for 20 min at 13,000 rpm. The clear supernatant was used to determine ascorbate and could be stored at −80 °C for several days.

### Determination of ascorbate

A modification of the bipyridyl method of Okamura (1980), as described by Knörzer, Burner & Boger (1996), was applied. (A) For the determination of AsA, a 125 μL sample of metaphosphoric acid extract was neutralised with 25 μL 1.5 M triethanolamine. After thorough mixing, 150 μL of sodium phosphate buffer (150 mM, pH 7.4) and 150 μL of H_2_O were added, and the mixture was further mixed. (B) For the determination of total ascorbate, a 125 μL sample was neutralised as above, mixed with 150 μL sodium phosphate buffer and 75 μL of 10 mM DTT and incubated for 15 min at room temperature to reduce the DAsA present in the extract. To remove excess DTT, 75 μL of 0.5 % (w/v) N-ethylmaleimide was added. Subsequently, the sample was mixed and incubated for at least 30 s at room temperature.

For subsequent procedures, both samples were treated identically. The following agents were added to the mixtures: TCA (300 μL, 10% (w/v)), phosphoric acid (300 μL, 44% (v/v) in 70% ethanol), and FeCl_3_ (150 μL, 3 % (w/v)). After mixing, the samples were incubated for 1 h at 37 ^0^C, and the absorption at 525 nm was recorded.

The concentration of DAsA was calculated by subtracting the AsA concentration measured in step A from the total ascorbate determined in step B. For standard curves of AsA or DAsA, the chemicals were prepared in 5% metaphosphoric acid.

### Statistical analysis

To evaluate the variation in duckweed growth parameters, enzyme activities and H_2_O_2_, MDA, AsA and DAsA contents, the control and treatment data (four and three independent biological replicates in growth experiments and enzyme assays, respectively) were analysed for normality of distribution (Shapiro–Wilk test) and homogeneity of variance (Levene’s test). Since the normality condition was met in each group, a one-way Analysis of Variance (ANOVA) followed by the *post hoc* Dunnett test (equal variances) or the Dunnett T3 test (unequal variances) was applied. If this condition was not met, a non-parametric Kruskal-Wallis one-way ANOVA followed by *post hoc* Dunn’s test was performed instead. A similar statistical procedure was employed to assess the variation in Pr content in *Lemna*. No data transformations were applied during statistical analysis. The analysis was conducted using SPSS Statistics 22.0 (IBM SPSS Statistics, Armonk, NY, USA).

## Results

### Praseodymium accumulation

Data on Pr accumulation are presented in Table 1. Accumulation of the lanthanide in the plant was concentration-dependent but increased non-linearly. A tenfold increase in Pr concentration in the medium from 0.01 to 0.1 mM resulted in a 56-fold increase. Further increases in Pr concentration from 0.2 to 0.4 mM resulted in less than a 50% increase in the plant. The highest BCF was calculated at 0.1 mM Pr.

**Table 1:** Praseodymium (Pr) concentrations in the external medium and in *L. minor* plants (*n* = 4), and bioconcentration factors (BCF).

PrCl_3_, mM	Pr (medium), g/L	Pr (plant), g/kg DW	BCF^*^, L/kg DW
0.01	0.0014	0.1507 ± 0.0296	107
0.1	0.0141	8.5688 ± 0.2923	608
0.2	0.0282	12.229 ± 0.2527	434
0.3	0.0423	13.986 ± 2.0300	331
0.4	0.0564	16.194 ± 0.4991	287

**Notes.**

* BCF = Pr (plant)/Pr (medium). DW, dry weight.

### The effect on the growth, hydrogen peroxide content and lipid peroxidation

Duckweed *L. minor* diminished frond and root growth rates when exposed to Pr (Fig. 1A). The first statistically significant (*p* < 0.05) decreases of frond and root growth rates compared to the control were observed at 0.2 and 0.1 mM of Pr, respectively. The inhibition of frond and root growth rates at the highest tested concentration of 0.4 mM equalled 36.8% and 93.8%, respectively. The early signs of chlorosis on the leaves were observed at 0.1 mM Pr, which gradually intensified at exposure concentrations ranging from 0.2 to 0.4 mM (Fig. 2).

**Figure 1:**
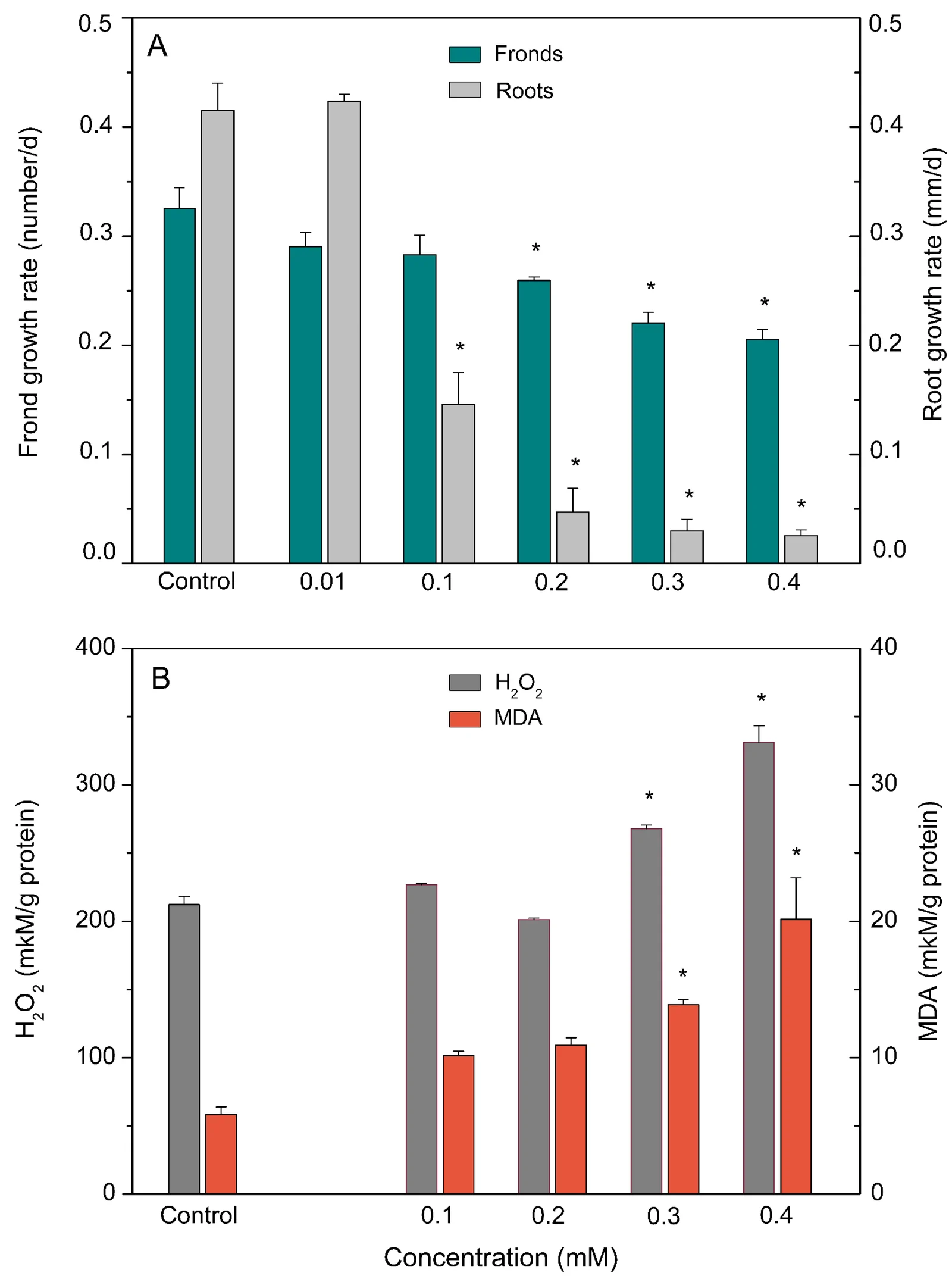
The effect of praseodymium on *Lemna minor* frond and root growth rates (A), and MDA and hydrogen peroxide content (B). The effect of praseodymium at 0.01, 0.1, 0.2, 0.3 and 0.4 mM on *Lemna minor* frond growth rate (in number d^−1^, *n* = 4), root growth rate (in mm d^−1^, *n* = 4), concentrations of hydrogen peroxide (in μM/g protein, *n* = 3) and MDA (in μM/g protein, *n* = 4) after 7-day treatment. Data are presented as mean ± SD. Asterisks indicate a statistically significant difference from the control at *p* < 0.05.

**Figure 2:**
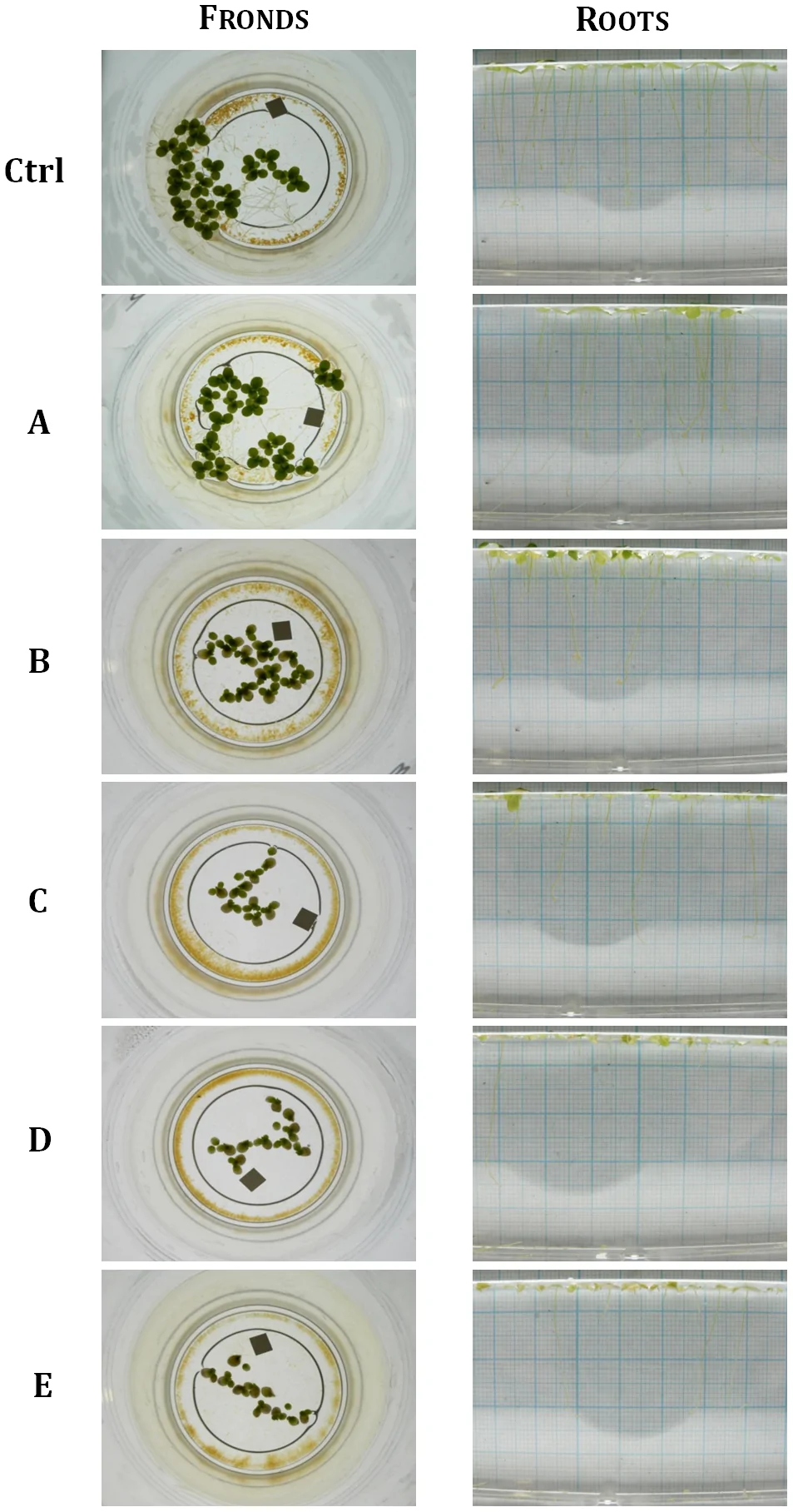
Duckweed *Lemna minor* fronds and roots treated with praseodymium. Duckweed *Lemna minor* (fronds and roots) exposed to control (Cntr), 0.01 (A), 0.1 (B), 0.2 (C), 0.3 (D) and 0.4 mM (E) of Pr.

The data on the concentration-dependent alterations of H_2_O_2_ and MDA contents are presented in Fig. 1B. Statistically significant (*p* < 0.05) increases in H_2_O_2_ concentration were measured in plants exposed to 0.3–0.4 mM Pr and were 268 ± 2.65 and 331 ± 11.9 µM/g protein (mean ± SD, n = 4) compared to the control of 212 ± 6.06 µM/g protein (n = 4). Similarly, statistically significant (*p* < 0.05) increases in MDA concentration were measured in plants exposed to 0.3–0.4 mM Pr. They were 13.9 ± 0.39 and 20.2 ± 0.30 µM/g protein (n = 3) compared to the control of 5.82 ± 0.58 µM/g protein (n = 3).

### Chlorophyll and carotenoid contents

Data on pigments are presented in Table 2. The lowest tested concentration, 0.01 mM Pr, did not induce statistically significant changes. Contents of chlorophylls decreased statistically significantly (*p* < 0.05) by approximately 38–52% at 0.1–0.4 mM Pr in relation to the controls. Within the same concentration range, carotenoid concentration decreased by approximately 20–30%.

### Changes in antioxidative enzymatic activities

Significant (*p* < 0.05) increase in CAT activity by 11–18% over the control (22.4 ± 0.85 µkat/g protein, n = 3) were measured in plants at 0.2–0.4 mM Pr (Fig. 2A). Similar, yet greater increase in activity of PPOD (peroxidase with pyrogallol as a substrate) by 10.4–58.8% over the control (27.2 ±1.16 µkat/g protein, n = 3) were measured at 0.1–0.4 mM Pr (Fig. 2C). Contrary, activity of GPOD (peroxidase fuelled by guaiacol as a substrate) decreased significantly by 41.2% below the control level (78.2 ± 3.53 µkat/g protein, n = 3) at the lowest tested Pr concentration of 0.1 mM (Fig. 2B). Then, the activity of GPOD augmented with the concentration increase in the range of 0.2–0.4 mM, however remaining below the control. APX activity in plants increased in the range of 0.1–0.4 mM Pr and was statistically significantly (*p* < 0.05) higher by 3.4–3.3 times compared to the control (4.10 ± 0.48 µkat/g protein, n = 4) at 0.3–0.4 mM Pr, respectively (Fig. 2D). DHAR showed similar induction profile as CAT, PPOD and APX (Fig. 2E). The activity of DHAR increased over the control (5.73 ± 0.12 µkat/g protein, n = 3) significantly by 4.7 and 13.6% at 0.3 and 0.4 mM Pr, respectively. GST activity increased gradually with the increase in Pr concentration and were significantly different from control level (1.45 ± 0.15 µkat/g protein, n = 3) at 0.1–0.4 mM Pr with the 76% activation compared to the control at 0.4 mM (Fig. 2F). AsA concentration did not show statistically significant differences compared to the control (0.896 ± 0.008 µkat/g protein, n = 4), while concentration of the DAsA decreased significantly below the control (0.547 ± 0.15 µkat/g protein, n = 4) at 0.1–0.4 mM Pr reaching DAsA activity lower by 4.3% compared to the control at 0.4 mM (Table 3).

**Table 2:** Content of chlorophylls (chl a and chl b) and carotenoids (CAR) in *L. minor* exposed to 0.01–0.4 mM praseodymium (in μg/g FW, *n* = 3). Asterisks indicate a statistically significant difference from the control at *p* < 0.05.

Pr	chl a	chl b	chl a + chl b	CAR
Control	0.370 ± 0.034	0.124 ± 0.009	0.494 ± 0.043	0.128 ± 0.010
0.01	0.347 ± 0.022	0.113 ± 0.004	0.460 ± 0.026	0.120 ± 0.004
0.1	0.231 ± 0.003^*^	0.074 ± 0.001^*^	0.305 ± 0.005^*^	0.102 ± 0.011^*^
0.2	0.224 ± 0.044^*^	0.075 ± 0.011^*^	0.299 ± 0.055^*^	0.096 ± 0.012^*^
0.3	0.191 ± 0.016^*^	0.063 ± 0.005^*^	0.254 ± 0.021^*^	0.087 ± 0.012^*^
0.4	0.176 ± 0.008^*^	0.067 ± 0.003^*^	0.243 ± 0.010^*^	0.090 ± 0.005^*^

**Table 3:** Concentrations of ascorboacetic acid (AsA), dehydroascorbic acid (DAsA), AsA+DAsA and AsA/DAsA in *L. minor* exposed to 0.1–0.4 mM praseodymium (in mg/g FW, *n* = 4). Asterisks indicate a statistically significant difference from the control at *p* < 0.05.

Pr, mM	AsA	DAsA	AsA + DAsA	AsA/DAsA
Control	0.8956 ± 0.0081	0.5704 ± 0.0062	1.4660 ± 0.0038	1.5704 ± 0.0303
0.1	0.8818 ± 0.0069	0.5595 ± 0.0019^∗^	1.4413 ± 0.0069^∗^	1.5760 ± 0.0142
0.2	0.8946 ± 0.0097	0.5547 ± 0.0033^∗^	1.4492 ± 0.0092	1.6129 ± 0.0225
0.3	0.8986 ± 0.0089	0.5471 ± 0.0058^∗^	1.4457 ± 0.0053^∗^	1.6428 ± 0.0321^∗^
0.4	0.9012 ± 0.0064	0.5458 ± 0.0025^∗^	1.4470 ± 0.0043^∗^	1.6511 ± 0.0189^∗^

## Discussion

The non-linear accumulation pattern and bioconcentration factor dynamics suggest that Pr uptake by *L. minor* reflects two overlapping processes: an initial high-affinity sequestration phase followed by a saturation- and toxicity-limited phase. The maximum BCF at lower concentrations (0.01–0.1 mM) is likely attributable to effective retention of Pr ions at negatively charged cell wall and apoplastic binding sites, which is consistent with findings of Xu et al. (2016) that, in *Spirodela polyrrhiza*, another duckweed species, a major fraction (65–69%) of accumulated Pr was associated with cellulose and pectin components. As external Pr concentration increased (0.2–0.4 mM), the decline in BCF despite continued tissue accumulation may reflect progressive saturation of these binding and uptake sites. In addition, higher Pr concentrations in our experiments coincided with root growth inhibition and oxidative damage, suggesting that the toxicity itself may have impaired further uptake *via* membrane transport pathways (see Martina, Ferroni & Marrocchino, 2025).

Various types of growth responses in *L. minor* have been reported in relation to lanthanides, including a hormetic effect or lack of response at lower concentrations, and progressive inhibition with increasing concentration (Weltje et al., 2002; Gjata et al., 2025). According to our data, praseodymium had no effect at 0.01 mM. It reduced the growth of fronds and roots in *L. minor* in a concentration-dependent manner in the range 0.1–0.4 mM, after 7-day exposure. To our knowledge, data on Pr toxicity in *Lemna* have not been reported in the literature. Other lanthanides, at the same initial (0.1 mM) concentration and exposure duration, were shown to affect frond growth in diverse ways: neodymium (Nd) and holmium (Ho) reduced growth rate by 1.3- and 16.7-fold, respectively, gadolinium (Gd) was ineffective, and cerium (Ce) showed stimulation (Gjata et al., 2025). The hormetic effect of Ce at 0.1–0.5 mM, irrespective of the anion in the cerium salt, was earlier reported by Zicari et al. (2018). The various sensitivities of *L. minor* to lanthanides suggest that lanthanides should not be considered a uniform group in terms of their physiological effects on aquatic plants, specifically on *Lemna*. Similarly, the biological effects of 12 REEs on the macrophytic alga *Nitellopsis obtusa* have been found to differ by two orders of magnitude (Manusadžianas et al., 2020). In contrast, the marine alga *Skeletonema costatum*, along with several other unicellular algae, showed a similar lanthanide (La) effect on viability; however, it could differentiate between La, Y (yttrium), and Sc (scandium) (Tai et al., 2010). Interestingly, Pr is a particular element in the 11-REE group due to an exceptionally high ratio of lethal to sublethal effects (Blaise et al., 2018).

*L. minor* leaves showed early symptoms of chlorosis at 0.1 mM concentration of Pr, signalling that metabolic processes are affected. This is in partial agreement with the appearance of chlorotic areas induced by Pr on the leaves of *S. polyrrhiza* (Xu et al., 2016). They observed that 26% of the leaf surface was damaged in the plants exposed for 20 days at 0.02 mM, whereas we did not observe the effect of Pr on *L. minor* growth or chlorosis at 0.01 mM after 7 days. This comparison suggests that different duckweeds are not equally susceptible to Pr. The lower sensitivity of *L. minor* to Ce ions has also been reported (Zicari et al., 2018).

Simultaneously with growth impairments in *L. minor* plants, Pr induced a dose-dependent increase in hydrogen peroxide levels, accompanied by enhanced lipid peroxidation, a typical symptom of cellular damage (Ippolito et al., 2010; Rajput et al., 2021). This damage occurs when the capacity of the natural antioxidant systems to detoxify activated oxygen species falls below the rate at which oxygen species are generated. REEs exert distinct effects on plant oxidative status. As reported by Gjata et al. (2025), *L. minor* exposed to seven lanthanides responded differently. For instance, Ce, Nd and Ho consistently increased MDA at the highest applied concentration (one mM) over a 12-day exposure period, suggesting severe oxidative damage to membrane components. In contrast, Gd, Dy (dysprosium), Yb (ytterbium), and Lu (lutetium) caused an initial increase in MDA within the first 3–7 days of exposure, followed by a decrease to the control level or below, indicating an adaptive or detoxifying response during prolonged exposure. This group of lanthanides has shown different sensitivity patterns at 10-fold lower concentration (0.1 mM), the lower end of the active Pr concentration range used in our study. In agricultural plants, however, Pr has been reported to reduce MDA content under cadmium stress in rice seeds (Zhou et al., 2009) and maize seedlings (Zhu et al., 2021).

In our study, 0.2–0.4 mM Pr induced significant changes in CAT activity, suggesting that CAT is involved in H_2_O_2_ breakdown into O_2_ and H_2_O, and in mitigating oxidative damage. The substrate for CAT is generatd by superoxide dismutases, the first enzymatic line of defence against stress-induced reactive oxygen species (ROS) (Habibi, 2014), catalysing the conversion of superoxide anions to H_2_O_2_ in chloroplasts and other organelles (see Halliwell & Gutteridge, 1985). When CAT activity is insufficient, class III extracellular peroxidases contribute to H_2_O_2_ dissipation (Hiraga et al., 2001). We assayed peroxidase activity using two substrates, guaiacol and pyrogallol, to capture substrate-specific responses. PPOD activity increased in *Lemna* exposed to Pr, paralleling H_2_O_2_ accumulation. In contrast, guaiacol-dependent GPOD activity decreased sharply, returning to control levels only at 0.4 mM Pr. However, unidirectional responses, strong increases in activity of POD fuelled by various substrates, have been observed in soybean cells under herbicide-induced oxidative stress (Knörzer, Burner & Boger, 1996. Elevated POD activity may promote lignification, strengthening cell walls as a defensive response to xenobiotics or stress (Bhattacharjee, 2012; Habibi, 2014). Transcriptional regulation of POD isoforms under stress varies across species and experimental conditions (Radić & Pevalek-Kozlina, 2010; Paradiso et al., 2016; Shi et al., 2025). POD isoforms’ responses to specific abiotic stress should be further investigated.

The photosynthetic electron transport chain is a significant source of ROS, where H_2_O_2_ can be detoxified in chloroplasts through the ascorbate–glutathione cycle using reducing power from NADPH (Foyer & Halliwell, 1976; Nakano & Asada, 1981; Rajput et al., 2021). AsA can also scavenge extracellular ROS, such as ozone (Polle, Wieser & Havranek, 1995). The oxidised forms of AsA, monodehydroascorbate and dehydroascorbate, are unstable in aqueous environments; thus, AsA deficiency can arise from increased AsA oxidation or impaired regeneration. In our experiments, ascorbate peroxidase, which uses AsA to reduce H_2_O_2_ to water, was significantly more active in Pr-exposed plants than in controls. Although APX induction is often associated with elevated AsA pools (De Gara, De Pinto & Arrigoni, 1997), we found that APX activity increased as the ascorbate pool was depleted, indicating AsA consumption during detoxification. Gjata et al. (2025) reported a marked increase in AsA content in *L. minor* treated with high concentration (one mM) of five out of seven tested Las (except Ho and Nd), consistent with an antioxidative response; lower AsA in Ho- or Nd-treated plants correlated with chlorosis and necrosis. Similarly, we observed chlorosis beginning at 0.1 mM Pr, alongside stable AsA levels.

We observed increased GST levels in *Lemna* following Pr exposure, suggesting an active role for GSTs in mitigating Pr-induced oxidative stress. GSTs reduce H_2_O_2_ *via* AsA-independent, thiol-mediated pathways (Vogelsang et al., 2024). Elevated H_2_O_2_ has been shown to strongly induce GST mRNA in soybean cells, supporting this interpretation (Levine et al., 1994). Increased GST activity was observed in plant leaves and roots of Cd-exposed *Pisum sativum* (Dixit, Pandey & Shyam, 2001) and *Phragmites australis* (Iannelli et al., 2002). Among heavy metals, a strong induction of GST activity occurred in Hg-treated root tips of *Hordeum vulgare* (Halušková et al., 2009). A similar pattern emerged in our study, in which GST activity increased with increasing Pr concentration, alongside inhibition of *Lemna* root growth. However, the GST activity can be disrupted by metals, potentially leading to detrimental toxic effects in plants (Teisseire & Guy, 2000). As reported by Letelier et al. (2006), copper (Cu) ions either alone or in the presence of AsA produce dose- and time-dependent inactivation of GST in rat liver cytosol. According to this reference, the major mechanism of Cu-ion-induced GST inhibition involves oxidation of its thiol groups rather than direct binding. Kang et al. (2022) revealed that REEs disrupt metabolic pathways, including those involving GST and acetaldehyde dehydrogenase. Hanana et al. (2021) shared a similar view, as changes in metallothionein levels, GST and cyclooxygenase activities indicated the cytotoxic effects of REEs in rainbow trout hepatocytes.

The antioxidant response observed in *L. minor* suggests activation of defence pathways in response to Pr exposure, but not complete protection against oxidative injury. Although CAT, PPOD, APX, DHAR and GST activities increased, H_2_O_2_ and MDA still accumulated significantly at higher Pr concentrations (0.3–0.4 mM), indicating that these responses were insufficient to fully counteract ROS production, membrane damage and growth impairments. Thus, the enzymatic changes should be interpreted as a stress response rather than evidence of successful detoxification. The decrease in GPOD activity at lower Pr concentrations, together with the increase in PPOD (Fig. 3), suggests that the peroxidase response was substrate specific. In addition, the stable AsA level combined with decreased DAsA (Table 3) may reflect altered turnover within the ascorbate-glutathione cycle, where oxidised ascorbate was rapidly processed, or the oxidised pool became depleted under sustained ROS pressure. Therefore, the overall biochemical pattern is more consistent with a partially effective yet ultimately insufficient antioxidant response at higher Pr exposure levels.

**Figure 3:**
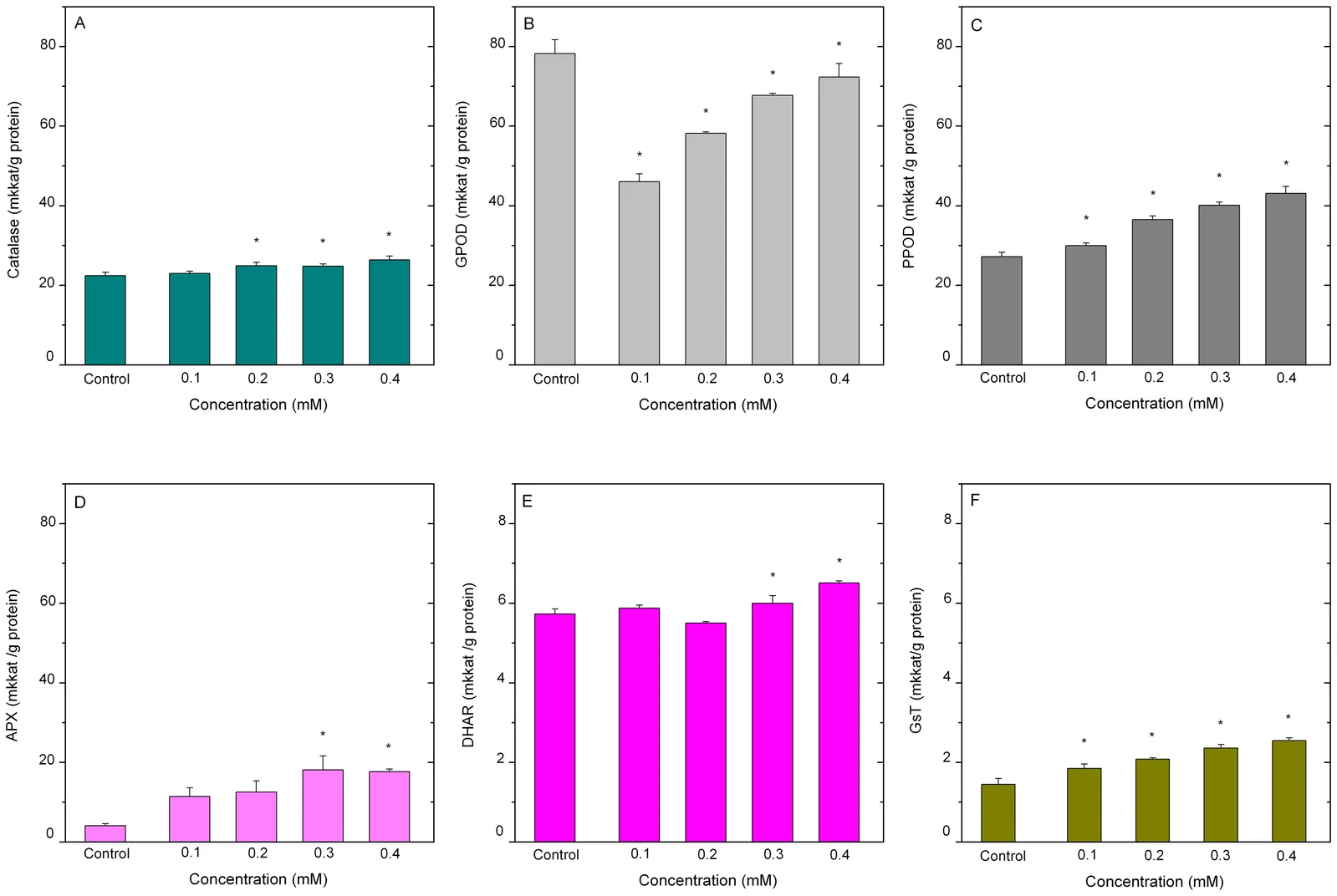
The oxidative stress enzyme activities in *Lemna minor* exposed to 0.1–0.4 mM praseodymium. The effect on *Lemna minor* oxidative stress enzyme activities (in μkat/g protein): CAT (A), GPOD (B), PPOD (C), APOX (D), DHAR (E) and GsT (F) (in mg/g protein, *n* = 3) after 7-day treatment of praseodymium at 0.1, 0.2, 0.3 and 0.4 mM. Data are presented as mean ± SD. Asterisks indicate a statistically significant difference from the control at *p* < 0.05.

A reduction in chlorophyll and carotenoid content, together with the visible chlorosis observed in exposed plants, supports the interpretation that Pr toxicity in *L. minor* extends beyond biochemical stress markers and also affects the photosynthetic apparatus. A similar pattern has been reported in *S. polyrrhiza* exposed to Pr, where increased MDA, reduced photosynthetic pigment content, inhibition of photosystem II, and chloroplast ultrastructural damage occurred together (Xu et al., 2016). Likewise, in *L. minor* exposed to other REEs, chlorophyll degradation was associated with growth inhibition and oxidative stress (Gjata et al., 2025). Therefore, the pigment data obtained in our study strengthen the conclusion that Pr affects not only redox status, but also functional plant performance.

## Conclusion

This study provided an integrated assessment of Pr toxicity in the aquatic macrophyte *Lemna minor*, combining Pr accumulation, growth and oxidative stress responses, antioxidant and biotransformation enzyme activities, and lipid peroxidation indicators. Exposure to increasing Pr concentrations resulted in augmentative concentration-dependent effects on plant development, demonstrating that *L. minor* is sensitive to Pr even under short-term laboratory conditions. Pr accumulated in *L. minor* tissues in a concentration-dependent manner, indicating effective uptake of this lanthanide from the surrounding medium. The observed accumulation coincided with growth inhibition, suggesting that internal Pr burdens contribute to the impaired physiological performance. Alterations in antioxidant and biotransformation enzyme activities, together with increased lipid peroxidation, indicate that Pr exposure disrupts cellular redox homeostasis and induces oxidative stress. Changes in enzymes associated with the ascorbate-glutathione cycle and detoxification pathways suggest their involvement in the plant’s response to Pr stress. However, these biochemical responses showed limited capacity to counteract oxidative damage at higher Pr levels. Overall, the combined evidence from growth, accumulation, and biochemical endpoints demonstrates that Pr can adversely affect *L. minor* physiology and fitness. These results expand the currently scarce experimental data on Pr toxicity in aquatic primary producers and can be helpful in ecotoxicological risk assessments of rare earth elements. Nevertheless, the present findings primarily define upper-bound toxicity responses of duckweed and reflect mechanistic effects at elevated exposures; therefore, they should not be directly extrapolated to the ecosystem level. Further studies focusing on longer exposure durations, environmentally relevant concentrations, and interactions with other stressors are needed to understand the ecological implications of Pr contamination in freshwaters.

## Supplemental Information

10.7717/peerj.21790/supp-1 Supplemental Information 1 Raw data of the *Lemna minor* responses to the praseodymium exposure On growth, pigment content, lipid peroxidation and enzyme activities.

## Additional Information and Declarations

### Data Availability

The following information was supplied regarding data availability: The raw data is available in the Supplemental File.
